# Host-protective effect of circulating pentraxin 3 (PTX3) and complex formation with neutrophil extracellular traps

**DOI:** 10.3389/fimmu.2012.00378

**Published:** 2012-12-13

**Authors:** Kenji Daigo, Takao Hamakubo

**Affiliations:** Department of Molecular Biology and Medicine, Research Center for Advanced Science and Technology, The University of TokyoTokyo, Japan

**Keywords:** PTX3, pentraxin, diagnosis, protein complex, anti-microbial protein, host-protection

## Abstract

Pentraxin 3 (PTX3) is a soluble pattern recognition receptor which is classified as a long-pentraxin in the pentraxin family. It is known to play an important role in innate immunity, inflammatory regulation, and female fertility. PTX3 is synthesized by specific cells, primarily in response to inflammatory signals. Among these various cells, neutrophils have a unique PTX3 production system. Neutrophils store PTX3 in neutrophil-specific granules and then the stored PTX3 is released and localizes in neutrophil extracellular traps (NETs). Although certain NET components have been identified, such as histones and anti-microbial proteins, the detailed mechanisms by which NETs localize, as well as capture and kill microbes, have not been fully elucidated. PTX3 is a candidate diagnostic marker of infection and vascular damage. In severe infectious diseases such as sepsis, the circulating PTX3 concentration increases greatly (up to 100 ng/mL, i.e., up to 100-fold of the normal level). Even though it is clearly implied that PTX3 plays a protective role in sepsis and certain other disorders, the detailed mechanisms by which it does so remain unclear. A proteomic study of PTX3 ligands in septic patients revealed that PTX3 forms a complex with certain NET component proteins. This suggests a role for PTX3 in which it facilitates the efficiency of anti-microbial protein pathogen clearance by interacting with both pathogens and anti-microbial proteins. We discuss the possible relationships between PTX3 and NET component proteins in the host protection afforded by the innate immune response. The PTX3 complex has the potential to be a highly useful diagnostic marker of sepsis and other inflammatory diseases.

## Introduction

The release of neutrophil extracellular traps (NETs), first reported in 2004 (Brinkmann, 2004), is one of the anti-microbial actions of neutrophils. NETs are mesh-like structures that contain DNA as a backbone, with anti-microbial proteins attached (Amulic and Hayes, 2011). NETs trap microbes and form an anti-microbial-protein-rich microenvironment (Medina, 2009).

Pentraxin 3 (PTX3) was reported as one of the NET component proteins (Jaillon et al., 2007). PTX3 is a member of pentraxin family and mainly acts as a soluble pattern recognition receptor (PRR) in the innate immune response (Bottazzi et al., 2010). In NETs, PTX3 may participate in microbial recognition by facilitating the trapping of microbes. The circulating PTX3 level is known to be increased in certain diseases, and PTX3 may predominantly play a critical role in host protection. Interestingly, proteomic identification of the circulating PTX3 interacting proteins revealed that PTX3 formed a complex with NET component proteins (Daigo et al., 2012). This finding implies that the NET component proteins are active in pathogen recognition and clearance by tethering with each other in NETs and bloodstream. PTX3 appears to be a key tethering molecule to enhance the actions of NETs component proteins. In this review, we will discuss the host-protective roles of PTX3 in relation to NETs component proteins.

## NETs

### Source, expression, and function

Neutrophils are the major player in the innate immune system response against microbial pathogen invasion. One of the anti-microbial activities of neutrophils is the extrusion of NETs (Brinkmann, 2004). NETs are formed upon the activation of neutrophils by factors such as IL-8, lipopolysaccharide (LPS), phorbol 12-myristate 13-acetate (PMA), bacteria, fungi, and activated platelets (Brinkmann, 2004; Clark et al., 2007; Fuchs et al., 2007). Neutrophil death as a result of the extrusion of NETs is called “NETosis,” which is a cell death pathway distinct from apoptosis or necrosis (Brinkmann and Zychlinsky, 2007; Steinberg and Grinstein, 2007). The release of NETs has also been reportedly observed without cell death (Yipp et al., 2012). Extracellular formations of this type are also observed in basophils and eosinophils (Schorn et al., 2012). NETs are mesh-like structures that consist of cellular DNA, along with bactericidal proteins, that reside in neutrophil granules and the nucleus. These proteins are connected to DNA fibers, and form a specialized microenvironment which facilitates the capture and killing of bacteria.

### The NET component proteins

Using a proteomic approach, Urban et al. identified 24 NET-associated proteins (Urban et al., 2009). These proteins are; nuclear components such as core histones; granular components such as neutrophil elastase (ELANE), lactotransferrin (LTF), cathepsin G (CTSG), myeloperoxidase (MPO), proteinase 3 (PRTN3), azurocidin 1 (AZU1), lysozyme C (LYZ), neutrophil defensins, and cytoplasmic proteins. In other proteins, histone H1, bactericidal permeability-increasing protein (BPI), pentraxin 3 (PTX3), and cathelicidin anti-microbial peptide (CAMP) are also defined as NET component proteins (Brinkmann, 2004; Jaillon et al., 2007; Lauth et al., 2009). Essentially all of these proteins possess anti-microbial activity.

## PTX3

### Genome

Breviario et al. identified PTX3 as one of the IL-1β -induced genes in human umbilical vein endothelial cells (HUVECs) (Breviario et al., 1992). The human PTX3 gene is located on chromosome 3q band 25, consists of 1861 base pairs, and is translated into 381 amino acids (Breviario et al., 1992). PTX3 belongs to the pentraxin family, which included the acute phase proteins C-reactive protein (CRP) and serum amyloid P-component (SAP). As PTX3 has a longer N-terminal domain, it is classified as a member of the long-pentraxin subfamily. Unlike the more common short pentraxins CRP and SAP, the *PTX3* gene is highly conserved across species (Garlanda et al., 2005). The *PTX3* gene consists of three exons, among which the first and second exons encode the signal sequence peptide and the N-terminal domain, and the third exon encodes the C-terminal domain. In the promoter region of the *PTX3* gene, a number of potential enhancer binding sequences (Pu-1, AP1, NF-κB, SP1, and NF-IL6) are located (He et al., 2007).

### Structure

After the processing of the signal sequence of the translated 1–17 amino acids, the mature PTX3 consists of two domains, i.e., the N-terminal domain (18–178 a.a.) and C-terminal domain (179–381 a.a.). The PTX3 C-terminal domain is a pentraxin-like domain, which is conserved among the pentraxin family with pentraxin signature (His-x-Cys-x-Ser/Thr-Trp-x-Ser). An N-linked glycosylation site (Asn220) is located in the C-terminal domain. In contrast to the C-terminal domain, the PTX3 N-terminal domain is a unique sequence unrelated to other proteins. The PTX3 protein forms an octamer via the inter-molecule disulfide bonds (Inforzato et al., 2008, 2010). Briefly, the N-terminal domain participates in the organization of a tetramer, and the C-terminal domain participates in the dimerization of the tetramer. Interestingly, the N-terminal tetramer formation has two states; a tetramer via the inter-disulfide bonds or non-covalent dimerization of the inter-disulfide-bonded dimer. This results in the asymmetric form of the full-length PTX3 (Inforzato et al., 2010).

### Expression pattern

PTX3 mRNA expression is induced by primary inflammatory signals in certain cells, such as myeloid dendritic cells (Doni et al., 2003), peripheral blood leukocytes (Alles et al., 1994), mononuclear macrophages/phagocytes (Alles et al., 1994; Goodman et al., 2000), vascular endothelial cells (Breviario et al., 1992; Lee et al., 1993), smooth muscle cells (Klouche et al., 2004), fibroblasts (Lee et al., 1993; Goodman et al., 2000), adipocytes (Abderrahim-Ferkoune et al., 2003), glial cells (Polentarutti et al., 2000), cumulus oophorus cells (Salustri et al., 2004), mesangial cells (Nauta et al., 2005), and synovial cells (Luchetti et al., 2000). Transcriptional activation of PTX3 in response to the pro-inflammatory cytokines TNFα and IL-1β is regulated by NF-κB binding site in the PTX3 promoter (Altmeyer et al., 1995; Basile et al., 1997). Other pathways also regulate PTX3 expression in a cell- and signal-dependent manner. In detail, please refer to the excellent reviews cited (He et al., 2007; Ortega-Hernandez et al., 2009; Deban et al., 2011; Inforzato et al., 2011).

The characteristic PTX3 expression pattern is observed in neutrophils. In mature neutrophils, the PTX3 protein is abundantly present in granules, but PTX3 mRNA expression is not detected. In contrast, PTX3 mRNA expression is observed in progenitor neutrophils, such as promyelocytes and myelocytes/metamyelocytes (Jaillon et al., 2007). As PTX3 protein expression is observed in both neutrophil precursors and mature neutrophils, it is considered that the PTX3 protein is produced during the course of neutrophil maturation and mature neutrophils store it for use-on-demand. Immunostaining revealed that PTX3 is present in neutrophil granules and that it colocalizes with lactoferrin (Jaillon et al., 2007; Savchenko et al., 2011), suggesting that PTX3 localizes to specific granules. The stored PTX3 in neutrophils is released upon *E. coli*, *S. aureus* or zymosan stimulation, as well as PMA, ionomycin or TNFα treatment (Jaillon et al., 2007; Savchenko et al., 2011; Daigo et al., 2012). PTX3 release is not induced by IL-1β or latex bead stimulation (Jaillon et al., 2007). The released PTX3 localizes to NETs and plays a non-redundant role in pathogen resistance. Thus, PTX3 in neutrophils plays a distinctive role in the innate immune response due to its rapid secretion, as well as by its unique pattern of ready-to-use expression and storage.

### Circulating levels

As the pentraxins CRP and SAP are well-known acute phase proteins, PTX3 may also be an acute phase biomarker. Under physiological conditions, the circulating PTX3 level is as low as approximately 2 ng/mL (Yamasaki et al., 2009). Recently, many studies on the circulating PTX3 level in clinical trials have been reported. These reports indicate that the PTX3 levels are significantly increased in certain infectious, cardiovascular, kidney, and female reproductive system diseases as well as other disorders (summarized in Table 1). In most cases, the PTX3 level correlates with both the severity and survivability of the disorder. In these diseases, the increases can reach up to 10~100 times the control level in severe inflammatory and infectious diseases such as sepsis. In the case of sepsis, the plasma PTX3 dramatically increases to a level of up to 100 ng/mL (Muller et al., 2001) and the increase correlates with mortality (Mauri et al., 2010).

**Table 1:** **Circulating PTX3 levels measurements in clinical trials**.

**Disease category**	**Diseases**	**PTX3 concentration and significance**	**References**
Physiological level		2.00 (1.95, 2.04)^a^	Yamasaki et al., 2009
Infectious diseases	Systemic inflammatory response syndrome (SIRS)	SIRS: 28.0 ± 5.6 Control: 1.04 ± 0.09^b^ *p* < 0.005	Muller et al., 2001
	Pulmonary tuberculosis (TB)	TB: 3.21	Azzurri et al., 2005
		Control: 0.98^c^ *p* < 0.0001	
	Sepsis	Sepsis: 26 (1, 202)	Hill et al., 2009
		Control: 6 (1, 12)^d^ *p* < 0.001	
	Febrile in the intensive/medium care unit (ICU/MC) or ward	In ICU/MC: 44.4 (13.6, 105.9)	De Kruif et al., 2010
		In ward: 14.2 (7.01, 25.1)	
		Control: 2.30 (1.66, 3.67)^d^ *p* = 0.01	
	Bacteremia	Non-survivor: 44.8 (10.7, 69.4)	Huttunen et al., 2011
		Survivor: 6.4 (3.4, 13.5)^d^ *p* < 0.001	
Cardiovascular diseases	Unstable angina pectoris (UAP)	UAP: 6.09 (4.34–7.85)	Inoue et al., 2007
		Control: 2.30 (2.03–2.55)^e^ *p* = 0.00003	
	Chronic heart failure (CHF)	CHF: 3.06 (2.38, 4.23)	Kotooka et al., 2008
		Control: 1.91 (1.35, 2.60)^d^ *p* = 0.001	
		Cardiac event: 6.0 (4.3, 9.3)	Ishino et al., 2008
		Event-free: 3.2 (2.0, 5.5)^d^ *p* < 0.001	
	Heart failure (HF)	Cardiac event: 6.22 (5.59)	Suzuki et al., 2008
		Event-free: 2.99 (2.95)^d^ *p* < 0.001	
		HF: 3.28 (1.51, 2.90)	Matsubara et al., 2011
		Non-HF: 2. 18 (1.51, 2.90)^d^ *p* < 0.001	
	Coronary artery disease (CAD)	CAD with inflammatory rheumatic disease (IRD): 1.96 ± 0.98	Hollan et al., 2010
		Control: 1.21 ± 0.59^b^ *p* < 0.001	
	Aortic valve stenosis (AS)	AS: 3.5 ± 1.9	Naito et al., 2010
		Control: 2.1 ± 0.8^b^ *p* < 0.05	
	Acute coronary syndrome (ACS)	ACS: 1.73 ± 0.82	Ustundag et al., 2011
		Control: 0.50 ± 0.39^b^ *p* < 0.001	
		ACS: 0.36 (0.225, 1.39)	Kume et al., 2011
		Control: 0.015 (0, 0.06)^f^ *p* < 0.0001	
	Hypertension	Anti-hypertensive mediation	Parlak et al., 2012
		Pre-treatment: 35.25 ± 5.45	
		Post-treatment: 0.14 ± 0.19^b^ *p* < 0.0001	
	Acute ischemic strokes	Non-survivor: 18.0 (8.2, 26.1)	Ryu et al., 2012
		Survivor: 6.4 (3.4, 11.8)^d^ *p* < 0.001	
	Giant cell arteritis (GCA)	GCA: 23.31 ± 4.06	Baldini et al., 2012
		Control: 3.97 ± 0.28^g^ *p* < 0.003	
Kidney diseases	Hemodialysis (HD)	HD: 3.03 ± 1.81	Malaponte et al., 2007
		Uremic patients: 2.34 ± 1.19	
		Control 1.03 ±0.4^b^ *p* < 0.001	
		HD: 1.87 (1.34, 2.50)	Xu et al., 2011
		Control: 1.11 (0.86, 1.51)^d^ *p* < 0.001	
		Renal transplant patients: 5.78 (1.09–20.36)	Argani et al., 2012
		HD group: 1.65 (0.24–7.89)^h^ *p* = 0.0001	
	Chronic kidney disease (CKD)	Stage 5 CKD: 5.7 (0.9, 64.3)	Tong et al., 2007
		Stage 3 to 4 CKD: 2.2 (0.4, 16.0)	
		Control: 1.8 (0.1, 9.1)^d^ *p* < 0.001	
		Stage 5 CKD: 5.3 (1.0, 58.0)	Suliman et al., 2008
		Control: 1.8 (0.1, 9.2)^d^ *p* < 0.001	
		CKD: 7.7 (1.8, 32.9)	Yilmaz et al., 2010
		Control: 1.3 (0.1, 2.7)^f^ *p* < 0.001	
		CKD: 3.80 ± 2.35	Nishi et al., 2011
		Control: 2.15 ± 0.93^b^ *p* < 0.0001	
		CKD with periodontitis: 6.3380 ± 2.74875	Pradeep et al., 2012
		CKD: 5.4100 ± 2.65296	
		Healthy: 1.8350 ± 0.75977^b^ *p* = 0.000	
Female reproductive system diseases	Preeclampsia (PE)	PE: 13.8 (3.9, 32.3)	Cetin et al., 2006
		Control: 2.2 (1.2, 3.8)^d^ *p* < 0.001	
		PE: 22.64 (18.56, 26.34)	Hamad et al., 2012
		Control: 13.17 (8.55, 16.54)^d^ *p* < 0.001	
	Pelvic inflammatory disease (PID)	PID: 9.3 ± 1.01	Chang et al., 2011
		Control: 2.27 ± 0.12^b^ *p* < 0.001	
	Polycystic ovary syndrome (PCOS)	PCOS: 1.0 ± 3.6	Aydogdu et al., 2012
		Control: 0.8 ± 0.8^b^ *p* = 0.021	
Others	Severe Psoriasis (sP)	sP: 2.84 ± 0.94	Bevelacqua et al., 2006
		Control: 1.22 ± 0.47^b^ *p* < 0.0001	
	Ulcerative colitis (UC) and crohn's disease (CD)	Active UC: 8.22 ± 5.48	Kato et al., 2008
		Active CD: 5.80 ± 3.59	
		Control: 1.76 ± 1.02^b^ *p* < 0.05	
	Obesity	Obesity: 0.99 ± 0.09	Miyaki et al., 2010
		Control: 0.63 ± 0.05^g^ *p* < 0.01	
	Central obesity in abdominal obesity patients	Central obesity: 3.00 ± 2.61	Shim et al., 2010
		Control: 1.33 ± 0.81^b^ *p* < 0.01	
	Severe traumatic brain injury (TBI)	non-survivors 9.95 (6.42)	Gullo Jda et al., 2011
		Survivors 5.46 (4.87)^b^ μg/mL *p* < 0.001	
	Obstructive sleep apnea (OSA)	Moderate-to severe OSA: 2.36 (1.79, 2.98)	Kasai et al., 2011
		Control: 1.53 (1.14, 2.04)^f^ *p* < 0.01	
	Schizophrenia (SZ)	SZ with the metabolic syndrome: 388.2 (504.1)	Beumer et al., 2012
		SZ: 430.4 (523.0)	
		Control: 213.6 (524.0)^d^ pg/mL *p* < 0.001	

PTX3 concentrations are shown in ng/mL, unless indicated.

a Geometrical mean (confidence interval).

b Mean ± SD.

c Geometrical mean.

d Median (interquartile range).

e Mean (95% confidence interval).

f Median (25th percentile, 75th percentile).

g Mean ± SEM.

h Median (Minimum-Maximum).

Although not included in Table 1, there are other infectious diseases, such as severe dengue virus infection (Mairuhu et al., 2005) and meningococcal disease (Sprong et al., 2009), in which the PTX3 levels are also increased. The PTX3 plasma concentration is increased in patients with acute myocardial infarction (Peri et al., 2000). During pregnancy, the serum PTX3 level slightly increases as the pregnancy progresses (Larsson et al., 2011). A higher PTX3 level is observed in preeclampsia (Cetin et al., 2006; Rovere-Querini et al., 2006). Finally, the serum PTX3 level is reported to be a biomarker for lung carcinoma (Diamandis et al., 2011). Thus, the circulating PTX3 level increases non-specifically in various infections and inflammatory disorders. For the purpose of diagnostic measurement, the dynamics of the PTX3 complex, such as the NET component proteins should be monitored (more details are discussed below).

### Function

PTX3 has been postulated to play a variety of roles in innate immunity, inflammatory regulation, and female fertility (Bottazzi et al., 2006; Garlanda et al., 2009; Inforzato et al., 2011; Cieslik and Hrycek, 2012). PTX3-knockout and transgenic mice studies have indicated that the predominant role of PTX3 occurs in host protection in the case of lung injury, infection, vascular damage, as well as certain other disorders (summarized in Table 2). Briefly, the resistance against pathogens such as *Aspergillus fumigatus*, *Paracoccidioides brasiliensis*, and *Klebsiella pneumoniae* has been reported (Garlanda et al., 2002; Diniz et al., 2004; Soares et al., 2006). In addition to its anti-pathogenic activity, PTX3 also has been shown to play a role in protecting against severe inflammatory reactions in animal models of sepsis (Dias et al., 2001), seizure-induced neurodegeneration (Ravizza et al., 2001) and acute myocardial infarction (Salio et al., 2008). In addition, PTX3 participates in extracellular matrix deposition. PTX3 is involved in the organization of hyaluronan in the viscoelastic matrix of cumulus oophorus (Scarchilli et al., 2007). It is considered that these functions of PTX3 are exhibited synergistically along with the binding of specific ligands (the details are provided in section “Ligands”).

**Table 2:** **Responses to certain disorders in PTX3-knockout and PTX3-transgenic mice**.

**Category**	**Experiment summary**	**Result summary**	**References**
Lung injury	Murine hepatitis virus strain 1 (MHV-1) infection	Causing greater severity of acute lung injury (ALI)^a^	Han et al., 2012
	Ventilator-induced lung injury (VILI)	Faster development of VILI^b^	Real et al., 2012
	LPS instillation	Causing greater severity of ALI^a^	Han et al., 2011
Vascular damage	Coronary artery ligation and reperfusion	Worsen heart damage^a^	Salio et al., 2008
	Atherogenic diet feed	Increased atherosclerotic lesion area in PTX3 and ApoE-double KO mice	Norata et al., 2009
	Ischemia and reperfusion of the superior mesenteric artery	Prevent tissue injury and mortality^a^	Souza et al., 2009
		Increased tissue injury and mortality^b^	Souza et al., 2002
Infection	LPS-induced endotoxemia	Increased survival ratio^b^	Dias et al., 2001
	CLP-induced sepsis	Increased survival ratio^b^	Dias et al., 2001
	Pulmonary infection by *Aspergillus fumigatus*	Decreased survival ratio^a^	Garlanda et al., 2002
	Pulmonary infection by *Klebsiella pneumoniae*	Faster lethality by a high inoculum administration^b^	Soares et al., 2006
		Delayed lethality by a mid-to-low inoculum administration^b^	Soares et al., 2006
	Murine cytomegalovirus (MCMV) infection	More susceptible to MCMV infection^a^	Bozza et al., 2006
	Influenza virus infection	More susceptible to influenza virus infection^a^	Reading et al., 2008
Others	Fas-deficient (lpr) C57BL/6 (B6) mice with mild lupus-like autoimmunity	Aggravate autoimmune lung disease in PTX3-KO B6^Lpr^ mice	Lech et al., 2011
	Kidney ischemia reperfusion injury	Less kidney injury and inflammation^a^	Chen et al., 2012
	Subcutaneous injection of Matrigel containing FGF2 and/or TSG-6	Abolishing of vascularization inhibition in PTX-KO mice	Leali et al., 2012
	Rolling interaction of PMNs in the mesenteric venules	Increased rolling interaction frequency^a^	Deban et al., 2010
	Sexual system	Subfertile^a^	Varani et al., 2002
	Kainate-induced seizures	More widespread seizure-related neuronal damage in the forebrain of PTX3-KO mice	Ravizza et al., 2001

a PTX3-knockout mouse study.

b PTX3-transgenic mouse study.

Of note, among the studies in PTX3-knockout and transgenic mice, there are some reports of an opposite effect of PTX3 on host-protection. In an intestinal ischemia and reperfusion model, Souza et al. reported an increased injury and lethality in the PTX3-transgenic mice that seemed to be associated with elevation of the TNFα concentration and aggravation of the inflammatory response (Souza et al., 2002). They also reported the suppression of tissue injury and lethality after ischemic and reperfusion in PTX3-knockout mice. PTX3 administration to these PTX3-knockout mice reversed this suppression (Souza et al., 2009). Other groups have also reported an adverse effect of PTX3 in acute ischemic lung injury (Chen et al., 2012) and ventilator-induced lung injury (Real et al., 2012). In the case of *Klebsiella pneumoniae* infection, faster lethality was observed when a higher inoculum was administered to PTX3-transgenic mice, but the lethality was conversely delayed when a middle or low inoculum was administered (Soares et al., 2006). Taking these bi-phasic functions of PTX3 in host-defense into account, more detailed accounts of the disease-specific mechanisms of PTX3 need to be elucidated to achieve useful clinical applications.

### Ligands

The multiple host-protective functions of PTX3 arise from the capacity for the recognition and binding to ligands. The reported PTX3 ligands are classified as follows: (1) complement components; (2) Fungi, bacteria, microbial components, and viruses; (3) selectin P; (4) extracellular matrix proteins and (5) growth factors (Presta et al., 2007; Mantovani et al., 2008; Deban et al., 2009; Moalli et al., 2011). Some of these ligands bind to PTX3 in a PTX3-domain specific manner, while others require full-length PTX3 for binding (Deban et al., 2009; Bottazzi et al., 2010).

1. PTX3 binds to certain select complement components, such as C1q (Inforzato et al., 2006), C4b-binding proteins (Braunschweig and Józsi, 2011), ficolins (Ma et al., 2009; Gout et al., 2011), mannose-binding lectin 2 (MBL) (Ma et al., 2011), factor H (Deban et al., 2008; Kopp et al., 2012), factor H-like protein 1 (Kopp et al., 2012) and factor H-related protein 1 (Kopp et al., 2012) for the regulation of the complement pathways in the innate immune response. The interaction of PTX3 and C1q elicits a dual consequence in the classical complement pathway. When C1q binds to immobilized PTX3, the classical complement pathway is activated; however, the binding of C1q to PTX3 in the fluid phase inhibits complement activation (Nauta et al., 2003). PTX3 can also activate the lectin pathway by binding to the ficolins and MBL. PTX3 enhances complement deposition by ficolin-2 on the *Aspergillus fumigatus* surface (Ma et al., 2009), and PTX3-MBL binding enhanced C4 and C3 deposition as well as the phagocytosis of *Candida albicans* (Ma et al., 2011). PTX3 is not only involved in complement activation, but also acts as a complement inhibitor to regulate excessive complement activation by binding to C4b-binding proteins and factor H. Please refer to the review by Doni et al. for more detail (Doni et al., 2012).
2. In the protection afforded against infection, PTX3 recognizes certain fungi, bacteria, microbial moieties, and viruses. PTX3 binds to microbial pathogens such as *Pseudomonas aeruginosa* (Garlanda et al., 2002), *Salmonella typhimurium* (Garlanda et al., 2002), *Aspergillus fumigatus* (Garlanda et al., 2002), and *Paracoccidioides brasiliensis* (Diniz et al., 2004). PTX3-knockout mice are susceptible to invasive pulmonary aspergillosis due to inappropriate Th1 and Th2-helper-cell-mediated resistance (Garlanda et al., 2002). Macrophages from PTX3-transgenic mice exhibit improved phagocytosis of *Paracoccidioides brasiliensis* as well as an enhancement of the production of nitric oxide (NO) (Diniz et al., 2004). PTX3 also binds to outer membrane protein A from *Klebsiella pneumoniae* (KpOmpA) in order to modulate the inflammatory response triggered by KpOmpA (Jeannin et al., 2005). PTX3 binds to cytomegalovirus and influenza virus type A for the inhibition of infection (Bozza et al., 2006; Reading et al., 2008). Upon binding to influenza virus, PTX3 exerts anti-viral activity by the inhibition of hemagglutination, the neutralization of virus infectivity and the inhibition of viral neuraminidase (Reading et al., 2008).
3. As an inflammatory modulator, PTX3 binds to selectin P. The N-linked glycosidic moiety of PTX3 contributes to the binding of selectin P, and this binding dampens neutrophil recruitment at the sites of inflammation (Deban et al., 2010). Importantly, in a model of acid-induced acute lung injury, both exogenous PTX3 and endogenously released PTX3 administration suppress neutrophil recruitment (Deban et al., 2010). This suggests a negative feedback role of PTX3 that dampens the excessive neutrophil recruitment via selectin P.
4. PTX3 takes part in extracellular matrix formation by binding to TNFα-induced protein 6 (TNFAIP6 or TSG-6) and inter-α-trypsin inhibitor (Iα I) (Salustri et al., 2004; Scarchilli et al., 2007; Ievoli et al., 2011). PTX3-knockout mice exhibit a defect in female fertility because of the defects in ovulation (Varani et al., 2002) and the organization of the cumulus oophorus extracellular matrix (Salustri et al., 2004). The PTX3-TSG-6 and PTX3-Iα I binding events are considered to be essential for the organization of hyaluronan in the viscoelastic matrix of cumulus oophorus (Inforzato et al., 2011; Moalli et al., 2011).
5. PTX3 binding to fibroblast growth factor 2 (FGF-2) regulates endothelial cell proliferation and angiogenesis, smooth muscle cell (SMC) activation, and intima thickening after arterial injury (Rusnati et al., 2004; Camozzi et al., 2005). PTX3-FGF2 binding can inhibit the proliferation and chemotactic activity of FGF2 in SMCs by interfering with the interaction of the FGF2 and FGF receptors (Camozzi et al., 2005).

Taking these results, it is clear that the protective effects of PTX3 are realized in coordination with specific PTX3 ligands. Therefore, we carried out a proteome-wide identification of PTX3 ligands and complexes in septic patient serum and plasma. PTX3 and its complex component proteins were immunoprecipitated by anti-PTX3 antibody-crosslinked magnetic beads, and the isolated fractions were subjected to shotgun proteomics analysis for label-free relative quantitation via spectral counting (Daigo et al., 2012). The identified proteins included the known PTX3 ligands such as C1q, ficolins, TSG-6, and Iα I, as mentioned above. Additionally, the ficolin-binding proteins of mannan-binding lectin serine protease 1 and 2 (MASP1 and MASP2) (Ma et al., 2009), and the TSG-6 binding proteins of the versican core protein (VCAN) and thrombospondin-1 (THBS1) (Salustri et al., 2004) were included in the proteins that were identified. As these proteins were identified in pooled normal human plasma with artificially spiked recombinant PTX3, these appear to be stable circulating PTX3 complexes. Nevertheless, the disease-specific dynamics of these binding levels need to be investigated further, as do the specific functions of these PTX3 complexes in sepsis.

## NET component proteins as PTX3 ligands: a newly recognized protective role

In the effort to identify the PTX3 ligand in septic patient fluids, a novel finding is that the NET component proteins were included (Daigo et al., 2012) (Table 3). A detailed investigation revealed that azurocidin 1 (AZU1) and myeloperoxidase (MPO) directly bind to PTX3. AZU1 and MPO belong to the NET component proteins (Urban et al., 2009) and exert bactericidal activity (Watorek, 2003; Klebanoff, 2005). AZU1 preferably binds to the PTX3 N-terminal domain, with a pattern of calcium ion dependency. In contrast to AZU1, MPO binds to both the PTX3 N-terminal and C-terminal domains, and does not require calcium ions. Further investigation of the PTX3-AZU1 interaction revealed that the AZU1 binding affinity to PTX3 was 22 ± 7.6 nM, and that AZU1 and PTX3 are partially co-localized in NETs (Daigo et al., 2012).

**Table 3:** **List of the NET component proteins and proteins belonging to the PTX3 complex**.

**NET component proteins**			**Proteomic identification of PTX3 complex in sepsis**
**Cellular localization**	**Protein name**	**Gene name**	
Granules	Neutrophil elastase	ELANE	
	Lactotransferrin	LTF	
	Azurocidin	AZU1	Yes
	Cathepsin G	CTSG	
	Myeloperoxidase	MPO	Yes
	Proteinase 3	PRTN3	
	Lysozyme C	LYZ	
	Neutrophil defensin 1 and 3	DEFA1 and 3	Yes (DEFA1)
	Pentraxin 3	PTX3	Target protein
	Bactericidal permeability-increasing protein	BPI	
	Cathelicidin anti-microbial peptide	CAMP	
Nucleus	Histone H1	H1F0	
	Histone H2A	H2A	Yes
	Histone H2B	H2B	
	Histone H2B-like	H2B	
	Histone H3	H3	Yes
	Histone H4	H4	Yes
	Myeloid cell nuclear differentiation antigen	MNDA	
Cytoplasm	S100 calcium-binding protein A8	S100A8	
	S100 calcium binding protein A9	S100A9	
	S100 calcium-binding protein A12	S100A12	
Cytoskeleton	Actin (beta and/or gamma 1)	ACTB, ACTG1	Yes
	Myosin-9	MYH9	Yes
	Alpha-actinin (1 and/or -4)	ACTN1, ACTN4	
	Plastin-2	LCP1	
	Cytokeratin-10	KRT10	Yes
Peroxisomal	Catalase	CAT	
Glycolytic enzymes	Alpha-enolase	ENO1	Yes
	Transketolase	TKT	Yes

**Figure 1:**
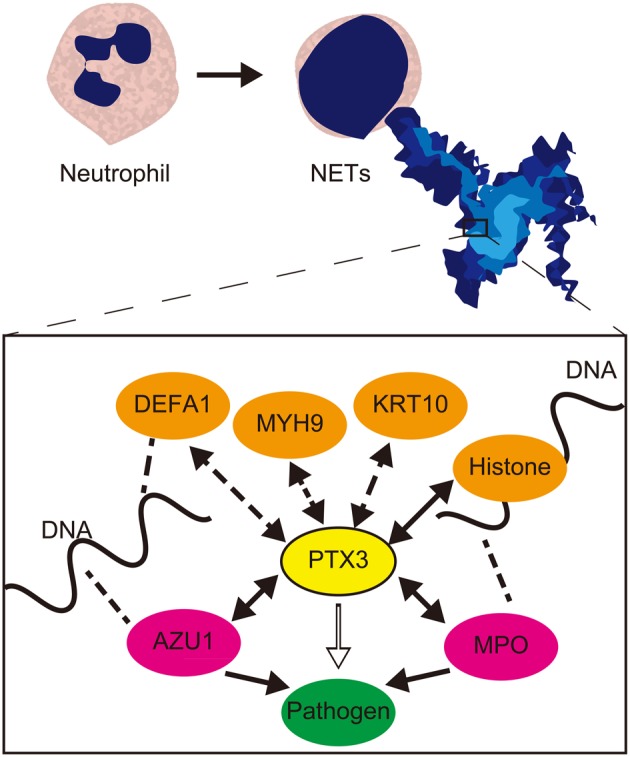
**Schematic relationship and role of PTX3 and NET component proteins in pathogen recognition and clearance.** NET component proteins which were identified as PTX3 complex (Daigo et al., 2012) are associated PTX3 in NETs. Among these, the confirmed direct interaction of AZU1 and MPO to PTX3, and formerly reported histone-PTX3 interaction (Garlanda et al., 2005) are designated by two-way arrows. These bindings facilitate pathogen clearance efficiency of AZU1 and MPO. The pathogen recognition and anti-pathogenic action are designated by open arrow and closed arrow in box, respectively. Two-way arrows with dashed lines designate other potential interactions to PTX3. The indirect association to DNA via histone or basic proteins such as DEFA1, AZU1, and MPO, which DNA associations are designated by dashed lines, maintains PTX3 localization in NETs. PTX3, pentraxin 3; DEFA1, neutrophil defensin 1; MYH9, Myosn-9; KRT10, Cytokeratin-10; AZU1, azurocidin 1; MPO: myeloperoxidase.

From these results, it is suggested that PTX3 may enhance the bactericidal efficiency of AZU1 and MPO in terms of both pathogen recognition and AZU1 and MPO binding (Figure 1). The mechanism by which PTX3 localizes in NETs has not yet been determined, but it is possible that PTX3 localization arises from an interaction with histones or the basic proteins AZU1, MPO, and defensin, along with a simultaneous association between these basic proteins and DNA (Figure 1). It is not clear at present whether the PTX3-AZU1 and PTX3-MPO binding events in the bloodstream take place within or outside of NETs. Either or even both of these are possible, and these complexes may be active in pathogen recognition and also involved in clearance. In septic patients, the plasma levels of AZU1 are increased, but do not significantly correlate with mortality (Berkestedt et al., 2010). As useful biomarkers of sepsis not yet available (Pierrakos and Vincent, 2010), the binding levels of PTX3-AZU1 and PTX3-MPO in septic plasma have the important potential to fulfill this purpose.

## Conclusion

Recent proteomic investigation of the circulating PTX3 complex components has revealed new and pivotal roles of PTX3 in the innate immune response, along with a pattern of binding to the NET component proteins. In NETs, PTX3 brings the NET component proteins into close proximity with the pathogens that PTX3 capture in order to enhance pathogen clearance. Also, in the bloodstream, PTX3 forms a complex with bactericidal proteins for the recognition and clearance of pathogens. These activities of PTX3 in concert contribute to the host-protective effect. In addition, the dynamic changes that occur in PTX3 and its complex proteins may become specific biomarker for severe inflammatory diseases.

### Conflict of interest statement

The authors declare that the research was conducted in the absence of any commercial or financial relationships that could be construed as a potential conflict of interest.
